# Relationship between the 10-Year Risk for Atherosclerotic Cardiovascular Disease and the Dietary Inflammatory Index among Korean Adults Based on the Seventh Korea National Health and Nutrition Examination Survey (KNHANES)

**DOI:** 10.1155/2020/8196798

**Published:** 2020-05-26

**Authors:** Ye-Na Lee, Purum Kang

**Affiliations:** ^1^College of Nursing, Korea University, Republic of Korea; ^2^College of Nursing, Woosuk University, Republic of Korea

## Abstract

Worldwide, atherosclerotic cardiovascular diseases (ASCVD) are the leading cause of death and are considered a major public health concern. Exposure to repeated inflammation may contribute to the development of ASCVD, and diet plays a vital role in inflammation. In this study, we explored the correlation between the dietary inflammatory index (DII) and the 10-year ASCVD risk in Korean adults. We used multistage, stratified sampling to analyze a representative sample of Korean adults aged 40-64 years from the 7^th^ Korea National Health and Nutrition Examination Survey data. Logistic regression was carried out to evaluate the association between 10-year high risk for ASCVD and dietary variables including DII. Participants were separated by quartiles, from Q1 to Q4, according to DII scores. Participants in the Q1 group had the lowest DII scores indicating a more anti-inflammatory diet. Participants in the Q4 group had the highest DII scores indicating more proinflammatory diets. Estimated risk of ASCVD results was categorized into the low-risk (less than 7.5% risk) and high-risk (greater than 7.5% risk) groups. In men, participants in the Q3 group had a risk for ASCVD of 1.20 times higher than the Q1 group participants and participants in the Q4 group had a risk of 1.34 times higher than the participants in the Q1 group. In women, ASCVD risk was not significantly associated with DII scores. These results provide systematically analyzed evidence for dietary interventions in ASCVD prevention efforts, especially in men.

## 1. Introduction

Globally, the proportion of the aging population is expanding at a rapid rate. Korea faces the prospect of having the largest aged population in the world [1]. Age-related reduction of physical function is a natural process; however, a variety of factors can expedite this process and in turn reduce an individual's capacity to perform activities of daily living. Cardiovascular disease is the leading cause of death in the elderly [2]. This condition is a result of both lifestyle and genetic factors and can reduce the quality of life for individuals as well as increase medical costs nationally [3]. Coronary artery disease, stroke, and peripheral artery disease are all conditions of atherosclerotic cardiovascular disease (ASCVD). In November 2013, the American Heart Association (AHA) and the American College of Cardiology (ACC) published a risk assessment tool for ASCVD [4]. The assessment calculates the risk of developing ASCVD within 10 years by evaluating factors such as age, blood pressure, smoking history, cholesterol levels, and diseases known to cause atherosclerosis. The assessment estimates the potential for disease development and offers targeted prevention strategies.

Inflammation is often a result of tissue damage [5]. When vascular inflammation occurs, it affects collagen and elastin, which in turn can damage the vascular endothelium that controls vascular contraction [6]. This may lead to permanent endothelial damage. The vascular endothelium plays a key role in regulating vascular contractility and is difficult to recover once the damage has occurred. When exposed to repeated inflammation, the vascular system is pathologically restructured over time, and the body's physiological feedback system may not respond properly [7]. This may contribute to the development of ASCVD and its related complications. Diet also plays an important role in inflammation levels, as shown by extensive research [8]. For example, recent studies show that folate intake may reduce inflammatory markers, and high-fiber diets may also be associated with reduced inflammation [9, 10]. Increased consumption of fruits and vegetables was found to reduce C-reactive protein and tissue plasminogen activator antigen (t-PA antigen), the inflammatory markers that are associated with vascular endothelial damage [11]. There is also a correlation between the consumption of food high in glucose and the elevated C-reactive protein levels [12]. Therefore, it can be concluded that dietary patterns and inflammation are closely related. A recent study showed that Koreans are consuming an increasing amount of sweetened beverages and fast food, which are known to elevate inflammatory markers, suggesting that dietary-related risk factors should be considered [13].

The dietary inflammatory index (DII) is a model derived from the results of 1943 articles and assesses the inflammatory effect of an individual's diet regardless of population and independent of specific dietary assessment methods [14]. Forty-five specific types of food, nutrients, and other bioactive compounds for consumption have been assessed for their capacity for changing the levels of specific inflammatory markers.

Regionally representative datasets based on diet surveys from 11 countries were used to establish comparative standards for each of the 45 parameters [15]. Several studies have focused on proinflammatory diets, as estimated by the DII, which have been associated with an increased risk of cardiovascular disease and an elevated risk of a first myocardial infarction [16]. Studies using data from Korean adults showed that DII is positively correlated with increased C-reactive protein levels [17]. However, there is still a need to identify the dietary factors associated with high risk for ASCVD in the Korean population. Therefore, this study investigated the relationship between the dietary related factors, including DII, and the 10-year ASCVD risk in Korean adults based on the 7^th^ Korea National Health and Nutrition Examination Survey (KNHANES) data. The study is aimed at enhancing the understanding of ASCVD and at suggesting methods of risk reduction. Moreover, the findings of this study could be used as an educational resource to reduce ASCVD and promote health programs.

## 2. Methods

### 2.1. Study Design

This is a descriptive, cross-sectional study that reviews data from the 7^th^ KNHANES to determine the relationship between the DII and the risk of ASCVD in Korean adults.

### 2.2. Data Collection

This study used data from the 7^th^ KNHANES conducted in 2016 and 2017 under the supervision of the Centers for Disease Control and Prevention under the Ministry of Health and Welfare. The KNHANES is an annual national research survey conducted since 1969 for direct public welfare. Its purpose is to provide representative and reliable national and municipal data on the health of the general population. The survey data are then used to guide health policies, develop and evaluate the goals of the Comprehensive National Health Promotion Plan, and design health promotion programs. The KNHANES uses data from the most recent population housing survey available at the time of sampling and can be used to extract representative samples of the target population (i.e., people over the age of 1 year) living in Korea. Data are collected via a health survey, screening survey, and nutrition survey.

In total, 16,277 individuals participated in the KHANES from 2016 to 2017. The participants ≥40 and ≤64 years old were included for the present study since the ASCVD risk calculation is specific for the age range 40 to 79 years and the food intake survey is conducted on participants under 64 years of age. Participants were excluded from the study if they were pregnant or had insufficient information to calculate the ASCVD risk or DII value. Finally, a total of 4185 participants were included in the analysis. The study design was approved by the Institutional Review Board of Korea University (IRB-2019-0174) and was considered exempt.

### 2.3. Calculation of the Dietary Inflammatory Index (DII)

The dietary inflammatory index (DII) was calculated using the food intake survey data obtained through a 24-hour recall method used in the KNHANES. Individual interviews were used to collect food intake data, and a nutrition research team consisting of two nutritionists visited households to investigate accurate information including spices, processed foods, and dietary supplements used in food with open question. To ensure validity, an interview was conducted a week later of medical examination, because there are many influencing factors that affect food intake such as fasting and drinking alcohol the day before the medical examination. The calculation of the DII was conducted based on a method previously reported on by Shivappa et al. [14]. A database consisting of diet surveys from 11 countries, including Korea, the USA, Australia, Bahrain, Denmark, India, Japan, New Zealand, Taiwan, and Mexico, was used to establish comparative standards for the parameters. The DII score was calculated based on the DII methods paper [14]. In brief, a standard mean for each parameter based on data from the global database was subtracted from individual scores from the KNHANES. The results were then divided by their standard deviation to generate *Z* scores. *Z* scores were converted to proportions to minimize the effect of outliers. The proportions were multiplied by two and then subtracted to achieve a symmetric distribution of inflammation scores for each DII factor. This information was used to obtain an overall DII score.

For the present study, we included the following 37 components to compute the DII score: carbohydrates, protein, total fat, monounsaturated fatty acids (MUFAs), polyunsaturated fatty acids (PUFAs), saturated fat, transfat, n-3 fatty acid, n-6 fatty acid, cholesterol, fiber, vitamin B_1_, vitamin B_2_, niacin, vitamin B_6_, vitamin B_12_, folic acid, vitamin A, vitamin C, vitamin D, vitamin E, b-carotene, iron, magnesium, selenium, zinc, flavan-3-ol, flavones, flavonols, flavonones, anthocyanidins, isoflavones, garlic, caffeine, onion, green and black tea, alcohol, and calories. The nutritional content data used in this study were from the Functional Ingredients Table (Rural Development Administration), Computer-Aided Nutritional Analysis (The Korean Nutrition Society), and data provided by the U.S. Department of Agriculture. Several previous studies have validated the use of the 37 parameters for the DII score [18–20]. For the assessment, positive scores indicate more proinflammatory diets and negative values are more anti-inflammatory.

### 2.4. ASCVD 10-Year Risk Calculation

According to the American College of Cardiology (ACC)/American Heart Association (AHA) guidelines, the 9 variables included in the ASCVD 10-year risk calculation are race, sex, age, total cholesterol, HDL cholesterol, blood pressure, diabetes diagnosis, treatment for hypertension, and smoking. Age was grouped by 10-year intervals. The variables were calculated using the pooled cohort equations (PCEs) through a web-based calculator (http://my.americanheart.org/cvriskcalculator) to compute the 10-year risk of ASCVD. The results were categorized into the low-risk (less than 7.5% risk), and high-risk (greater than 7.5% risk) groups [4].

### 2.5. Data Analysis

KNHANES is a cross-sectional survey of the Koreans which assessed the overall health and nutrition. This dataset was designed as a complex, stratified, multistage, and probability-cluster sampling which has been certified as appropriate for representative statistics. Thus, it is a nationally representative sample of individuals from the noninstitutionalized Korean population. Thus, we used a complex sampling method that considers the characteristics of the raw data to analyze the KNHANES data. To analyze the KNHANES data, we used a complex sampling method that considers the characteristics of the raw data. Analysis of the mixed-sample design utilized stratification, niche, and weights provided by the Centers for Disease Control and Prevention to increase the representativeness and accuracy of variable estimates. The collected data was analyzed using SPSS v. 22.0 software (IBM Corp., Armonk, NY, USA). *p* value of <0.05 was considered significant for all statistical tests. The general characteristics of the participants are summarized with descriptive statistics. The 10-year risk of ASCVD and the DII in Korean adults were compared between groups by the analysis of variance and *χ*^2^ test. Logistic regression analyses were performed to identify the association between the quartile of DII and the ASCVD 10-year risk according to sex.

## 3. Results

### 3.1. General Characteristics of the Study Population

The general characteristics of the study population are shown in Table 1. Among 4185 participants, 1712 were men and 2473 were women. The mean age was 52.51 ± 0.17 years. Participants were separated by quartiles, from Q1 to Q4, according to DII scores. Participants in the Q1 group had the lowest DII scores indicating a more anti-inflammatory diet. Participants in the Q4 group had the highest DII scores indicating more proinflammatory diets.

### 3.2. Effect of DII and Dietary-Associated Factors on the ASCVD Risk Group in Men and Women

Characteristics of the study participants according to sex in quartiles of the DII are presented in Table 2. In both men and women, subjects with higher DII scores were characterized by lower household income, lower subjective body image, poor subjective health status, current smoker, diabetes mellitus diagnosis, poor treatment for hypertension, and ASCVD high-risk group compared to subjects with lower DII scores (*p* < 0.001, respectively). We observed statistically significant positive associations between the quartiles of the DII scores and the ASCVD high-risk group (≥7.5) in men with 36.9% in Q1 and 45.1% in Q4 (*p* < 0.001). In women, the quartiles of the DII scores were significantly positively related to the ASCD high-risk group (≥7.5) with 4.7% in Q1 and 4.8% in Q4 (*p* < 0.001).

To evaluate the effects of DII scores on the risk of ASCVD in Korean men and women, a multivariate logistic regression analysis was performed (Table 3). In men, subjective health status had a significant effect on ASCVD risk (*p* < 0.001). According to subjective health status, the ASCVD risk for the “poor” group was 1.75 times higher than that for the “good” group, and the risk in the “moderate” group was 1.32 times higher than that in the “good” group. DII scores according to quartiles were significantly associated with ASCVD risk (*p* < 0.05). Participants in the Q3 group had a risk for ASCVD of 1.20 times higher than the Q1 group participants, and participants in the Q4 group had a risk 1.34 times higher than the participants in the Q1 group. In women, subjective health status had a significant effect on the risk of ASCVD (*p* < 0.001). However, ASCVD risk was not significantly associated with DII scores (Table 3).

## 4. Discussion

This study is aimed at analyzing multistage, stratified sampling data representative of the population to determine 10-year ASCVD risk among Koreans aged 40-64 years and at classifying risk according to dietary variables. The results show that elevated DII scores in Korean men are positively associated with a higher risk for ASCVD over a 10-year period. In women, subjective health status is associated with ASCVD risk, but DII scores are not an accurate predictor of 10-year ASCVD risk.

Understanding and classifying factors associated with a higher risk of ASCVD is important to guide prevention efforts. The 10-year ASCVD risk calculation was developed for estimating risk for ASCVD and classifying high-risk groups using multiple factors such as age, race, HDL-C, TC, SBP, sex, smoking status, and treatment for hypertension or diabetes mellitus [4]. Since cardiovascular disease is closely related to lifestyle [21], we evaluated the relationship between 10-year ASCVD risk and DII scores, which reflect dietary lifestyle.

The DII score is based on the relationship between systemic inflammatory biomarkers and diet and indicates the overall inflammatory potential of a diet using 45 parameters [14]. In this study, the quartile of DII reflected differences in demographic variables that could affect the quality of the diet, such as subjective body image [22], household income [23], and subjective health status [24]. Thus, those vulnerable demographic characters seem to be related to unhealthy dietary habit, contributing to the potential systemic inflammation state. Moreover, since chronic inflammation also has an important role in the pathogenesis of ASCVD [25], the DII is used to reflect an individual's amount of potential inflammatory exposure through diet and therefore also reflects the risk for ASCVD. These results are consistent with previous research that finds a close relationship between systemic inflammation and ASCVD, including atherosclerosis [26].

Our study showed a difference in ASCVD risk according to sex. Specifically, the DII score was associated with the high-risk group in Korean men, whereas there was no significant association between the DII score and the risk in Korean women. This is consistent with a previous study that found a difference in risk for cardiovascular disease according to sex [27]. It has also been reported that women tend to be less susceptible to environmental stress, such as oxidative stress, than men [28]. One factor influencing the difference may be related to sex hormones. Estrogen is a potent sexual steroid hormone in women that is known for its antioxidant and anti-inflammatory effect [29]. Thus, a systemic inflammatory state related to dietary habits may have less of an effect on women. Another explanation for the difference may be associated with a poorer lifestyle. In the present study, men had a high prevalence of unhealthy lifestyle habits such as smoking. Thus, lifestyle modification, including dietary intervention, is needed to control and prevent ASCVD in high-risk groups of men.

This study has some limitations. First, the 10-year ASCVD risk score was developed based on Western sample populations; thus, it may not be completely suitable for Koreans. Second, there were potential limitations to the accuracy of the DII score because DII calculation was conducted by a self-reported questionnaire. In addition, information was not available for some DII parameters. Furthermore, since this was a cross-sectional study, it is difficult to determine causality. Despite these limitations, this is the first study to confirm that the DII score is positively correlated with the 10-year ASCVD risk using a well-defined, nationally representative sample in Korea.

## 5. Conclusion

This study evaluated the association of quartile of DII and 10-year ASCVD risk in Korean adults. In conclusion, we demonstrated that the quartile of DII is associated with the 10-year ASCVD risk in Korean men, providing evidence for link between the highly proinflammatory diet identified as DII and the risk of ASCVD. In addition, these results provide systematically analyzed evidence for dietary interventions in ASCVD prevention efforts, especially in men.

## Figures and Tables

**Table 1:** General characteristics of participants from the 7^th^ Korea National Health and Nutrition Examination Survey.

Variables		Total		Men		Women	
		(*n* = 4185)	Weighted % (SE)	(*n* = 1712)	Weighted % (SE)	(*n* = 2473)	Weighted % (SE)
Age, years (SE)		52.51 (0.17)		52.14 (0.20)		52.87 (0.20)	
Household income	Lowest	492	10.0 (0.7)	192	9.2 (0.9)	300	10.7 (0.8)
	Lower middle	982	21.5 (0.9)	366	19.6 (1.1)	616	23.3 (1.1)
	Upper middle	1190	29.2 (1.0)	475	28.2 (1.3)	715	30.1 (1.1)
	Highest	1518	39.4 (1.4)	678	42.9 (1.7)	840	35.9 (1.5)
Subjective body image	Very skinny	114	2.9 (0.3)	74	4.2 (0.6)	40	1.5 (0.3)
	Slightly skinny	403	9.8 (0.6)	210	12.1 (1.0)	193	7.6 (0.6)
	Moderate	1678	40.4 (0.9)	699	40.6 (1.3)	979	40.2 (1.3)
	Slightly obese	1605	38.4 (0.9)	644	38.0 (1.4)	961	38.8 (1.1)
	Very obese	385	8.5 (0.5)	85	5.0 (0.6)	300	11.9 (0.7)
Subjective health status	Good	1109	28.3 (0.8)	517	31.7 (1.2)	592	25.1 (1.0)
	Moderate	2224	55.7 (0.9)	879	54.5 (1.4)	1345	56.8 (1.2)
	Poor	705	16.0 (0.7)	241	13.7 (0.9)	464	18.1 (0.9)
Current smoker, %		749	21.4 (0.9)	642	38.6 (1.5)	104	4.6 (0.5)
Diabetes mellitus diagnosis, %		312	7.0 (0.5)	157	8 (0.7)	155	6.1 (0.6)
Treatment for hypertension, %		859	18.7 (0.7)	417	21.6 (1.1)	442	15.8 (0.9)
Systolic blood pressure, mmHg (SE)		119.47 (0.34)		121.35 (0.45)		117.65 (0.42)	
Fasting glucose, mmol/L (SE)		102.21 (0.44)		105.45 (0.65)		99.09 (0.52)	
Total cholesterol, mmol/L (SE)		200.95 (0.67)		198.50 (0.97)		203.34 (0.85)	
HDL cholesterol, mmol/L (SE)		51.04 (0.23)		47.24 (0.31)		54.74 (0.29)	
DII	Q1	1046	25.5 (0.9)	507	29.7 (1.3)	539	21.5 (1.0)
	Q2	1046	25.1 (0.8)	447	25.7 (1.2)	599	24.5 (1.0)
	Q3	1046	25.1 (0.8)	375	22.5 (1.2)	671	27.8 (1.1)
	Q4	1047	24.2 (0.9)	383	22.1 (1.3)	664	26.3 (1.1)
ASCVD risk, % (SE)		4.93 (0.10)		7.46 (0.16)		2.47 (0.06)	

Abbreviations: SE: standard error; HDL: high-density lipoprotein; DII: dietary inflammatory index; ASCVD: atherosclerotic cardiovascular disease.

**Table 2:** Characteristics of the study participants according to sex in quartiles of the DII scores.

Variables		Men (*n* = 1712)					Women (*n* = 2473)				
		Q1	Q2	Q3	Q4	*p* value	Q1	Q2	Q3	Q4	*p* value
*n*		507	447	375	383		539	599	671	664	
Age, years (Mean, SE)		52.33, 0.32	51.86, 0.33	52.22, 0.37	52.11, 0.36	0.73	53.46, 0.33	53.21, 0.34	52.67, 0.30	52.27, 0.33	0.03^∗^
DII (Mean, SE)		−3.63, 0.05	−1.16, 0.03	0.08, 0.04	3.48, 0.07	<0.001^∗∗^	−3.57, 0.05	−1.16, 0.03	0.79, 0.03	3.55, 0.03	<0.001^∗∗^
Household income (*n*, weighted %)	Lowest	25, 4.9%	33, 6.7%	46, 10.4%	88, 16.8%	<0.001^∗∗^	42, 7.6%	76, 10.9%	81, 10.1%	101, 13.8%	<0.001^∗∗^
	Lower middle	89, 14.6%	115, 24.3%	84, 20.4%	78, 19.9%		113, 19.1%	140, 22.2%	184, 26.4%	179, 24.4%	
	Upper middle	148, 30.2%	123, 26.7%	112, 30.1%	92, 25.5%		157, 28.6%	164, 29.0%	203, 32.3%	191, 29.9%	
	Highest	245, 50.3%	176, 42.3%	133, 39.0%	124, 37.9%		227, 44.7%	219, 38.0%	201, 31.1%	193, 31.8%	
Subjective body image (*n*, weighted %)	Very skinny	17, 3.5%	17, 3.9%	16, 4.5%	24, 5.4%	<0.001^∗∗^	11, 1.7%	10, 1.2%	7, 1.1%	12, 1.5%	<0.001^∗∗^
	Slightly skinny	55, 11.0%	58, 12.4%	48, 13.6%	49, 11.8%		37, 6.1%	48, 8.0%	55, 8.2%	53, 7.6%	
	Moderate	213, 40.7%	174, 40.7%	161, 41.5%	151, 39.5%		229, 44.3%	249, 41.7%	267, 40.2%	234, 40.2%	
	Slightly obese	199, 40.1%	181, 39.6%	135, 36.8%	129, 34.7%		208, 38.4%	237, 39.7%	250, 36.3%	266, 38.8%	
	Very obese	23, 4.7%	17, 3.4%	15, 3.6%	30, 8.7%		54, 9.5%	55, 9.3%	92, 14.1%	99, 11.9%	
Subjective health status (*n*, weighted %)	Good	193, 40.0%	141, 33.0%	105, 30.1%	78, 21.0%	<0.001^∗∗^	130, 25.5%	148, 26.1%	176, 26.1%	138, 22.8%	<0.001^∗∗^
	Moderate	246, 51.7%	229, 53.8%	202, 55.9%	202, 57.7%		300, 58.4%	309, 54.1%	374, 57.4%	362, 57.3%	
	Poor	43, 8.3%	63, 13.2%	52, 14.0%	83, 21.3%		89, 16.1%	130, 19.9%	107, 16.5%	138, 19.9%	
Current smoker (*n*, weighted %)		148, 29.8%	153, 36.8%	152, 41.7%	189, 49.5%	<0.001^∗∗^	13, 2.6%	22, 3.9%	23, 3.0%	49, 8.5%	<0.001^∗∗^
Diabetes mellitus diagnosis (*n*, weighted %)		39, 7.4%	44, 8.1%	33, 8.2%	41, 8.4%	<0.001^∗∗^	28, 5.5%	40, 5.6%	43, 6.4%	44, 6.5%	<0.001^∗∗^
Treatment for hypertension (*n*, weighted %)		127, 22.7%	106, 19.6%	90, 21.4%	94, 22.5%	<0.001^∗∗^	94, 15.0%	105, 15.5%	117, 15.8%	126, 16.9%	<0.001^∗∗^
Systolic blood pressure, mmHg (Mean, SE)		121.5, 0.80	119.99, 0.74	121.33, 0.90	122.75, 0.92	0.11	117.53, 0.73	116.60, 0.78	118.60, 0.78	117.73, 0.75	0.35
Fasting glucose, mmol/L (Mean, SE)		104.92, 1.06	105.31, 1.53	105.40, 1.37	106.24, 1.46	0.91	97.98, 0.78	98.33, 0.10	99.77, 0.98	100.0, 1.10	0.31
Total cholesterol, mmol/L (Mean, SE)		199.91, 1.57	197.47, 1.97	197.69, 2.13	198.61, 1.99	0.78	201.64, 1.76	200.60, 1.66	205.90, 1.53	204.58, 1.77	0.07
HDL cholesterol (Mean, SE)		48.24, 0.58	46.55, 0.53	46.75, 0.62	47.20, 0.60	0.15	55.12, 0.62	55.20, 0.63	54.31, 0.57	54.4, 0.51	0.61
ASCVD high-risk group (*n*, weighted %)		222, 36.9%	201, 34.2%	178, 41.1%	209, 45.1%	<0.001^∗∗^	31, 4.7%	30, 4.0%	36, 4.5%	41, 4.8%	<0.001^∗∗^

Abbreviations: Q: quartile; DII: dietary inflammatory index; SE: standard error; HDL cholesterol: high-density lipoprotein cholesterol; ASCVD high-risk group: atherosclerotic cardiovascular disease high-risk group. ∗∗p values < 0.001 are considered significant.

**Table 3:** Weighted logistic regression analysis of factors affecting high risk of 10-year ASCVD according to sex.

			OR	95% CI
Men	DII^∗^	Q1	Reference	
		Q2	0.89	0.66-1.21
		Q3	1.20	0.86-1.67
		Q4	1.34	0.10-1.8
	Subjective health status^∗∗^	Good	Reference	
		Moderate	1.32	1.03-1.69
		Poor	1.75	1.23-2.47

Women	DII	Q1	Reference	
		Q2	0.75	0.39-1.44
		Q3	0.94	0.51-1.73
		Q4	0.98	0.52-1.83
	Subjective health status^∗∗^	Good	Reference	
		Moderate	1.82	0.92-3.57
		Poor	3.09	1.49-6.40

Abbreviations: OR: odds ratio; DII: dietary inflammatory index. ^∗∗^*p* values < 0.001 are considered significant. ^∗^*p* values < 0.05 are considered significant.

## Data Availability

The data used to support the findings of this study are available from the corresponding author upon request.
